# Evaluation of Reported Adverse Drug Reactions in Antibiotic Usage: A Retrospective Study From a Tertiary Care Hospital, Malaysia

**DOI:** 10.3389/fphar.2018.00809

**Published:** 2018-08-20

**Authors:** Ann L. Arulappen, Monica Danial, Syed A. S. Sulaiman

**Affiliations:** ^1^Discipline of Clinical Pharmacy, School of Pharmaceutical Sciences, Universiti Sains Malaysia, Georgetown, Malaysia; ^2^Pharmacy Department, Hospital Pulau Pinang, Georgetown, Malaysia; ^3^Clinical Research Center, Hospital Pulau Pinang, Georgetown, Malaysia

**Keywords:** adverse drug reactions, vancomycin, trimethoprim/sulfamethoxazole, penicillin, skin

## Abstract

Adverse drug reaction (ADR) primarily caused by many drugs including antibiotics. At present, the incidence and pattern of ADR caused by antibiotics have remained as neglected area in Malaysia. This study was conducted to determine the incidence and analyze the pattern of ADR caused by antibiotics among patients in a tertiary care hospital. It is a 2-year retrospective observational study conducted at Hospital Pulau Pinang, Malaysia. All eligible patients who had antibiotic prescribed belonging to any age group either from outpatient or inpatient that had experienced ADR was included in this study. The outcomes were measured with the aid of Naranjo’s and Hartwig’s scales. The incidence of the ADRs among patients prescribed with antibiotics in Hospital Pulau Pinang is about 1.1%. Vancomycin and Trimethoprim/Sulfamethoxazole both are considered to be the major contributors to ADR incidences. The skin was the most affected organ by ADRs followed by gastrointestinal system. Most of the severe ADRs were caused by Penicillin. The causality relationship of all the severe reactions was mostly probable. General Medicine unit had reported the highest number of ADRs caused by antibiotics. The common manifestations of ADRs are acute kidney injury and exanthem. In addition, majority of the ADRs caused by antibiotics were reversible. A large multicenter study is suggested to confirm the present findings.

## Introduction

One of the major threats in the healthcare system is the adverse drug reaction (ADR), which profusely affects the health, transience, and the quality of life especially among the hospitalized patients (Lazarou et al., 1998; Pirmohamed et al., 1998, 2004; Davies et al., 2009). In many countries, ADRs rank among the top 10 leading causes of mortality. Therefore, there is a need to study ADRs seriously to create awareness among patients and to motivate health care professionals in the hospital to report ADRs as in to minimize the risk (Hurwitz, 1969; Hepler, 2003; Morris and Cantrill, 2003; Schnipper et al., 2006; Knudsen et al., 2007).

A study conducted in Malaysia among the pediatric population revealed that anti-infective for systemic use (61.9%) was the most common therapeutic group reported for ADRs in children below 2-years-old (Rosli et al., 2016). Another 8 years retrospective study conducted in Malaysia revealed that anti-infective agents were among the drugs caused various ADRs (35%) (Ibrahim et al., 2013). Pharmacovigilance activities are regulated by the National Center for Adverse Drug Monitoring, a division within the National Pharmaceutical Regulatory Agency (NPRA), which was established under Drug Control Authority (DCA) in Malaysia. Malaysian Adverse Drug Reactions Advisory Committee (MADRAC) provides DCA with important information pertaining to local and international drug safety issues and plays a role as advisor to DCA on risk management and risk communication following effective assessment of the benefit-risk profile of drugs (Malaysian Guidelines for the Reporting and Monitoring of Adverse drug Reactions, 2017). MADRAC revealed that 50% of the top 10 drugs most commonly reported for cutaneous ADR in the year 2008 belong to the antibiotic group (Ding et al., 2010).

Although there are various studies underlying the ADR effects among patients prescribed with antibiotics, but the data on the ADR incidence and pattern have remained as neglected area. Thus, this study was conducted with the aim to determine the incidence and analyze the pattern of the ADRs among patients prescribed with antibiotics in Hospital Pulau Pinang, Malaysia. This study will help to gain insight on the number of patients that are affected with ADR as a consequence from antibiotic use and provide the much needed data to prevent reoccurrence of the ADR associated with those specific antibiotics especially in different patients and further reduces the impact on the patients’ quality of life.

## Materials and Methods

### Study Settings and Design

This was a 2-year retrospective observational study using patients’ medical records conducted at Hospital Pulau Pinang, Malaysia. A total of 648 patients’ medical records from January 2015 till December 2016 were retrieved via the MADRAC registry. All eligible patients’ records belonging to any age group prescribed with an antibiotic by the prescriber either from outpatient or inpatient whom had experienced ADR were included in this study. Validated data collection form was used in obtaining patients’ clinical and the socio-demographics data. The total population of patients whom visited Hospital Pulau Pinang was 15,712 for the duration of 2 years. Written approval was obtained from the Medical Research and Ethics Committee (NMRR-16-1740-32642), Ministry of Health Malaysia prior to the embankment of the study. No personal identifiers of the patients were collected during the study duration. The data were accessed anonymously.

### Verification of Scoring System

Naranjo’s scale was used for the causality assessment in this study (Naranjo et al., 1981). The first scoring of the scale was given by the reporter whom could be any of the healthcare professionals. Subsequently, the pharmacist whom handled the ADR submissions to the MADRAC re-scored the scale for further confirmation and verified the details being reported. Additionally, Hartwig’s scale was used for the severity assessment scale in this study (Hartwig et al., 1992). The first scoring of the scale was given by the reporter whom could be any of the healthcare professionals. However, the researcher verified the scores given by the reporter with the information written in the bed head ticket (BHT). Any queries or doubts arises during the process were clarified immediately with the reporter.

### Statistical Analysis

The data were entered and analyzed using the Statistical Package for Social Sciences (SPSS) base version 22.0; SPSS, Inc., Chicago, IL, United States. Descriptive data analysis was used for the analysis of this study. The normally distributed continuous variables are summarized in mean and standard deviation while the non-normalized are expressed as median and inter quartile range. For categorical variables, frequencies and percentage were tabulated. Two-sided *p*-values of less than 0.05 were considered statistically significant.

## Results

During the study period, a total of 648 patients’ records that had documented the event of ADR were screened at the study site. Out of it, only 175 reactions were reported caused by antibiotics. And all 175 reports met the eligibility criteria and subjected for further analysis. None was excluded. Baseline clinical and socio-demographic characteristics of the patients enrolled in the study are depicted in **Table 1**.

**Table 1:** Patients’ baseline socio-demographic and clinical characteristics (*N* = 175).

Variable	Mean + SD	No. (%)
**Gender**
Male		92 (52.6)
Female		83 (47.4)
**Ethnicity**
Malay		80 (45.7)
Chinese		69 (39.4)
Indian		23 (13.1)
Other		3 (1.7)
**Age (years)**	45 + 22	
≤18 (Pediatrics)		28 (16)
19–64 (Adults)		122 (69.8)
≥65 (Geriatrics)		25 (14.2)
**Department**
In-patient		148 (84.6)
Out-patient		27 (15.4)
**Reason for admission**
Infection related		96 (54.9)
Non-infection related		52 (29.7)
**Marital status**
Married		125 (71)
Unmarried		50 (29)
**Education level**
Educated		139 (79)
Uneducated		36 (21)
**Smoking**
Non-smokers		93 (53)
Active + ex-smokers		82 (47)


**Table 2** describes the various analyses of the ADRs caused by antibiotics. Skin manifestations deemed to be the highest organ to be affected by ADRs followed by gastrointestinal system. Penicillins are proven to be the main culprit in causing the most of the adverse reactions followed by Cephalosporins. General Medicine unit had reported the highest number of ADRs. This study revealed that all the ADRs were moderate reactions in terms of severity and believed to have probable causality the most. Precisely, majority of the ADRs caused by antibiotics were reversible.

**Table 2:** Various analyses of the reported adverse drug reactions caused by antibiotics (*N* = 175).

Type of analysis	Number of reported ADR (*n*)	Percentage of ADR (%)
**Analysis of organ affected by ADR**		
Skin	84	48
Gastrointestinal	19	10.9
Hematologic	16	9.1
Hepatic	13	7.4
Immunologic	5	2.9
Musculoskeletal	4	2.3
Neurologic	8	4.6
Ophthalmic	8	4.6
Others	3	1.7
Renal	15	8.5
**Analysis of antibiotic categories causing ADR**		
Aminoglycoside	4	2.3
Carbapenem	10	5.7
Cephalosporin	36	20.6
Glycopeptide	21	12
Lincosamide	3	1.7
Macrolide	9	5.2
Others	29	16.6
Oxazolidinone	3	1.7
Penicillin	56	32
Quinolone	2	1.1
Tetracycline	2	1.1
**Analysis of departments that reported the ADR**		
Cardiology	6	3.4
Cardiothoracic	6	3.4
Skin	9	5.2
Gastroenterology	2	1.1
General medicine	69	39.4
Hematology	5	2.9
Nephrology	25	14.3
Obstetrics and gynecology	2	1.1
Oncology	2	1.1
Orthopedics	1	0.6
Pediatrics	21	12
Respiratory	19	10.9
Surgical	8	4.6
**Analysis of severity assessment of the ADR**		
Mild	58	33.1
Moderate	101	57.7
Severe	16	9.2
**Analysis of causality assessment of the ADR**		
Definite	16	9.1
Possible	74	42.3
Probable	85	48.6
Doubtful	0	0
**Analysis of reversibility of the ADR**		
Yes	172	98.3
No	3	1.7


The manifestations of the ADRs were further illustrated in **Table 3**. Exanthem has accounted as the common manifestation in quite a number of patients. And the subsequent common manifestation seen in this study was acute kidney injury.

**Table 3:** Details on the manifestations of adverse drug reactions caused by antibiotics.

Manifestations of adverse drug reactions caused by antibiotics	Number of reported ADR (*n*)	Percentage of ADR (%)
Acute kidney injury	15	8.6
Anaphylactic shock	3	1.7
Arthralgia	1	0.6
Angioedema	3	1.7
Agitation	1	0.6
Chills and rigors	1	0.6
Coagulopathy	7	4
Diarrhea	13	7.5
Exanthem	44	25.1
Erythema multiforme	2	1.1
Exanthematous pustulosis	1	0.6
Exfoliative dermatitis	1	0.6
Encephalopathy	1	0.6
Erythema nodosum	1	0.6
Face flushing	2	1.1
Hallucination	4	2.3
Hyperbilirubinemia	2	1.1
Nausea and vomiting	6	3.4
Pancytopenia	1	0.6
Periorbital swelling	8	4.6
Pruritus	11	6.3
Raised liver enzymes	11	6.3
Rashes	12	6.8
Seizure	2	1.1
Stevens–Johnson syndrome	6	3.4
Thrombocytopenia	8	4.6
Urticaria	6	3.4
Upper and lower limbs swelling	2	1.1


Further analysis of the antibiotics causing ADRs based on the various pharmacological categories is depicted in **Table 4**. Vancomycin and Trimethoprim/Sulfamethoxazole are known to cause the highest number of ADRs equally. The manifestations of ADRs are acute kidney injury and exanthem respectively.

**Table 4:** Details on the antibiotics caused the adverse drug reactions.

Antibiotic category	Antibiotic name	Number of reported ADR (*n*)	Percentage of ADR (%)
Penicillin	Amoxicillin	13	7.4
	Amoxicillin/clavulanic acid	18	10.6
	Ampicillin	3	1.7
	Ampicillin/sulbactam	8	4.6
	Benzyl penicillin	3	1.7
	Cloxacillin	10	5.7
	Piperacillin/tazobactam	1	0.6
Cephalosporin	Cefazolin	3	1.7
	Cefepime	6	3.4
	Cefoperazone	7	4
	Cefoperazone/sulbactam	2	1.1
	Cefotaxime	1	0.6
	Cefuroxime	6	3.4
	Ceftaroline	2	1.1
	Ceftazidime	4	2.3
	Ceftriaxone	3	1.7
	Cephalexin	2	1.1
Glycopeptide	Daptomycin	1	0.6
	Vancomycin	20	11.4
Carbapenem	Doripenem	1	0.6
	Ertapenem	1	0.6
	Imipenem	3	1.7
	Meropenem	5	2.9
Macrolide	Azithromycin	1	0.6
	Clarithromycin	2	1.1
	Erythromycin	6	3.4
Aminoglycoside	Amikacin	2	1.1
	Gentamicin	2	1.1
Lincosamide	Clindamycin	3	1.7
Oxazolidinone	Linezolid	3	1.7
Quinolone	Ciprofloxacin	2	1.1
Tetracycline	Doxycycline	2	1.1
Others	Trimethoprim/sulfamethoxazole	20	11.4
	Rifampicin	9	5.2


**Table 5** portrayed the top six antibiotics causing the ADRs and their manifestations respectively.

**Table 5:** Top six antibiotics caused the adverse drug reactions and their respective manifestations.

No.	Antibiotics caused adverse drug reactions	Manifestations of adverse drug reactions	No. ADR reported (*n*)	No.	Antibiotics caused adverse drug reactions	Manifestations of adverse drug reactions	No. ADR reported (*n*)
1	Vancomycin	Acute kidney injury	10	3	Amoxicillin	Exanthem	4
		Exanthem	4			Rashes	3
		Face flushing	2			Periorbital swelling	2
		Urticaria	1			Anaphylactic shock	1
		Pruritus	1			Agitation	1
		Thrombocytopenia	1			Diarrhea	1
		Rashes	1			Erythema multiforme	1
	Trimethoprim/sulfamethoxazole	Exanthem	9	4	Cloxacillin	Exanthem	2
		Stevens–Johnson syndrome	2			Rashes	2
		Thrombocytopenia	2			Anaphylactic shock	1
		Acute kidney injury	2			Pruritus	1
		Periorbital swelling	1			Chills and rigors	1
		Pancytopenia	1			Periorbital swelling	1
		Urticaria	1			Upper and lower limbs swelling	1
		Erythema multiforme	1			Nausea and vomiting	1
2	Amoxicillin/clavulanic acid	Diarrhea	6	5	Rifampicin	Exanthem	4
		Exanthem	3			Raised liver enzymes	2
		Stevens–Johnson syndrome	2			Nausea and vomiting	1
		Raised liver enzymes	2			Pruritus	1
		Periorbital swelling	2			Arthralgia	1
		Erythema nodosum	1		
		Pruritus	1		
		Exfoliative dermatitis	1		


## Discussion

To the best of our knowledge, this is the first study which analyzed the pattethe pattern of ADRs caused by antibiotics in Malaysia. This study provides the much needed data about the incidence rate of antibiotics causing ADRs especially in a tertiary care hospital. The incidence rate of antibiotics causing ADRs in this study was found to be comparatively low when compared to other studies (Ding et al., 2010; Priyadharsini et al., 2011; Shamna et al., 2014; Dhar et al., 2015). It accounted about 1.1% (*n* = 175) out of the total population which had been reported past 2 years. The incidence rate was lower probably due to the effective intervention of respective clinical pharmacists at the study site. Also, we were informed that the health care professionals in this hospital receive constant updates from the Medication Safety team formed merely within Hospital Pulau Pinang, thus, keeping them updated of the latest events related to drug safety via their routine publication of bulletins.

In this study, the predominance of male sex for ADRs which is similar to those studies conducted elsewhere (Jose et al., 2008; Dhar et al., 2015). According to the data obtained from the record office, majority of the admitted patients was male throughout the study period. Analysis of the age wise distribution showed the predominance of adult patients followed by pediatric patients and lastly geriatrics. Probably adult patients were more prone to antibiotic ADRs due to age related pharmacokinetics and pharmacodynamics changes and the presence of co-morbid illnesses and multiple drugs along with infectious diseases.

On the other hand, the most affected organ system by ADRs due to antibiotics is skin which is parallel with another study (Ibrahim et al., 2013) followed by gastrointestinal, hematologic, renal, hepatic, neurologic, ophthalmic, musculoskeletal, and others. Contrary to our study, other studies conducted elsewhere (Shamna et al., 2014; Dhar et al., 2015) claimed that the most affected organ system was the gastrointestinal system. Out of the many types of skin reactions, exanthem was the most commonly accounted type of adverse reaction in this study. Another two studies (Ding et al., 2010; Priyadharsini et al., 2011) concluded that the most common manifestation observed were maculopapular eruption and urticaria, respectively.

Another noteworthy finding of the current study was the distribution of antibiotic classes that caused ADRs. Penicillins was the most accounted antibiotic category causing ADRs which is similar to other studies conducted elsewhere (Priyadharsini et al., 2011; Rosli et al., 2016), followed by Cephalosporins, others precisely Trimethoprim/Sulfamethoxazole and Rifampicin, Glycopeptides, Carbapenems, Macrolides, Aminoglycosides, both Lincosamides and Oxazolidinones and lastly Quinolones and Tetracyclines. Penicillins was the most accounted antibiotics category causing ADRs compared to the rest of the pharmacological categories is because the usage of Penicillins was the highest throughout the study period compared to Cephalosporins. This study also revealed Vancomycin and Trimethoprim/Sulfamethoxazole are both antibiotics with highest number of incidences of ADRs. Vancomycin as it is known to be a nephrotoxic drug, deemed to cause most of the ADRs as in acute kidney injury and the usage was high consistent with the high occurrence of Methicillin-resistant *Staphylococcus aureus* (MRSA) infection. Trimethoprim/Sulfamethoxazole usage was also high due to high prevalence of *Stenotrophomonas maltophilia* isolates which resulted from heavy usage of Carbapenems. Trimethoprim/Sulfamethoxazole were reported to have most of skin reactions, especially exanthem. We suppose this exanthem reaction was due to presence of sulfonamides in Trimethoprim/Sulfamethoxazole.

The highest quantity of ADR reports were received from General Medicine followed by Nephrology, Pediatrics, Respiratory, Skin, Surgical, both Cardiology and Cardiothoracic, Hematology, Gastroenterology, Obstetrics and Gynecology and Oncology, and last of all were from Orthopedics. In this study, most of the reported ADRs were from General Medicine is because the department used ranges of antibiotics tremendously right from the low end to the high end throughout the study period for both prophylaxis and treatment purposes. Furthermore, the Infectious Unit is grouped under General Medicine Department in this hospital.

Of all the reported ADRs, moderate reactions accounted the most and followed by mild and severe reactions as per the Hartwig’s scale. Other studies (Jose et al., 2008; Stavreva et al., 2008; Priyadharsini et al., 2011; Shamna et al., 2014; Dhar et al., 2015) also disclosed comparable findings as ours. This study revealed that none of the severe ADRs were fatal provided all the cases were given intensive medical care accordingly. Most of the severe ADRs were caused by Penicillins and the most common manifestation of severe ADR is Stevens–Johnson syndrome (SJS). The one death reported in severe category was purely due to severe head injury post-trauma. The adults were accounted the most with severe ADRs compared to the rest of the age groups. Male gender was identified to be more prominent than female in this sub-analysis.

Moreover, the causality assessment of reported ADRs as per the Naranjo’s scale and revealed that mostly were probable, then possible and limited reactions were definite. There were no ADR reports grouped as unlikely. These findings are analogous as Stavreva et al. (2008); Priyadharsini et al. (2011), Shamna et al. (2014), and Dhar et al. (2015). The cases were categorized as definite after “rechallenging” with the respective drugs. All the definite cases were given medical care accordingly and none of them is fatal. Most of the definite ADRs were caused by Rifampicin and the most common manifestation of definite ADR is exanthem. Perhaps, the adults were accounted the most with definite ADRs compared to the rest of the age groups. Female gender was identified to be more prominent than male in this sub-analysis.

In this study, it is noted that majority of the total reported ADRs were reversible. Limited numbers of reactions were irreversible. All the irreversible reactions were reported from General Medicine. The reason behind the irreversibility is because all the patients involved died even before the ADRs were treated. Indeed, those patients died because of severe head injury post-MVA. The utmost common manifestation of the irreversibility reaction is acute kidney injury and caused by vancomycin and gentamicin. Another antibiotic namely, Trimethoprim/Sulfamethoxazole had caused irreversible thrombocytopenia. None of the reported irreversible ADRs is due to inappropriate doses or frequencies. All the irreversible ADRs were experienced by adults and mostly were male patients.

## Conclusion

The incidence rate of antibiotics causing ADRs in this study was found to be as low as 1.1%. Thus, educational interventions on the main causes of ADRs, patient monitoring, and appropriate prescribing policy should be deployed to both prevent ADRs and minimize their impact, where they do occur. Dissemination of information related to ADRs to both health care professionals and patients has the potential to improve awareness and subsequently heightens the patients’ quality of life.

### Strength and Limitations

The strength of the study is that the information collected was directly from the experienced health care professionals itself. This group of professionals receive constant updates from the Medication Safety team formed merely within Hospital Pulau Pinang, thus, keeping them updated of the latest events or news related to drug safety via their routine publication of bulletins. Being a study from a single center in a high burden country with ADRs caused by antibiotics, the findings of the present study should be interpreted with the major limitation of small number of patients enrolled. However, Hospital Pulau Pinang is a center for referral especially for the northern region population and also being a second largest hospital in Malaysia. Thus, the usage of antibiotics is tremendously high and involves huge variation of antibiotics. Nevertheless, a multicenter study with a large sample size is needed to confirm the findings of the current study. On the other hand, there is possibility of under reporting especially by the newly joined health care professionals such as house physicians and provisionally registered pharmacists.

## Author Contributions

AA and MD conception and design, analysis interpretation of the data, and statistical expertise. AA collection and assembly of data and drafting of the article. SS critical revision of the article for important intellectual content and final approval of the article.

## Conflict of Interest Statement

The authors declare that the research was conducted in the absence of any commercial or financial relationships that could be construed as a potential conflict of interest.
